# Accelerated pharmaceutical protein development with integrated cell free expression, purification, and bioconjugation

**DOI:** 10.1038/s41598-018-30435-4

**Published:** 2018-08-10

**Authors:** Dominique Richardson, Jaakko Itkonen, Julia Nievas, Arto Urtti, Marco G. Casteleijn

**Affiliations:** 1 Drug Research Program, Division of Pharmaceutical Biosciences, Faculty of Pharmacy, University of Helsinki, Helsinki, Finland; 2 Division of Pharmaceutical Chemistry and Technology, Faculty of Pharmacy, University of Helsinki, Helsinki, Finland; 3 School of Pharmacy, Faculty of Health Sciences, University of Eastern Finland, Kuopio, Finland; 4 Institute of Chemistry, St Petersburg State University, Petergoff, St Petersburg, Russian Federation

## Abstract

The use of living cells for the synthesis of pharmaceutical proteins, though state-of-the-art, is hindered by its lengthy process comprising of many steps that may affect the protein’s stability and activity. We aimed to integrate protein expression, purification, and bioconjugation in small volumes coupled with cell free protein synthesis for the target protein, ciliary neurotrophic factor. Split-intein mediated capture by use of capture peptides onto a solid surface was efficient at 89–93%. Proof-of-principle of light triggered release was compared to affinity chromatography (His_6_ fusion tag coupled with Ni-NTA). The latter was more efficient, but more time consuming. Light triggered release was clearly demonstrated. Moreover, we transferred biotin from the capture peptide to the target protein without further purification steps. Finally, the target protein was released in a buffer-volume and composition of our choice, omitting the need for protein concentration or changing the buffer. Split-intein mediated capture, protein *trans* splicing followed by light triggered release, and bioconjugation for proteins synthesized in cell free systems might be performed in an integrated workflow resulting in the fast production of the target protein.

## Introduction

In traditional biotechnology, cells are utilized for a variety of different processes^1^, and more recently during the eras of genetics and molecular biology, one important process is recombinant protein production^2–5^. For pharmaceutical protein production and screening the use of cells is a common practice and the state-of-the art^6–8^. The bioprocess for recombinant protein production is divided in an upstream and a downstream process: cells are fermented before and during protein production (upstream) and the product is either secreted from the cells into the cultivation media, or cells are lysed and the cell lysates processed further (downstream). Downstream processes may include: separation of soluble and insoluble cell debris and media components, protein purification, protein formulation (including concentrating), bioconjugation, protein-bioconjugate purification, and protein refolding. Depending on the protein and the needs either all, some, or just one of these aforementioned downstream processes are required^9^. Proteins are prone to degradation due to many triggers, such as pH, salt concentration, organic solvents, shearing, interaction with surfaces and interfaces (including protein aggregates), lyophilisation, humidity levels, protein concentration, and temperature changes. Therefore, these processes must to be designed carefully to produce intact and functional proteins.

Regarding the physicochemical basis for protein purification we can identify low, medium and high-resolution methods^10^. Low resolution methods are based on precipitation due to differences in solubility, for example in different ammonium sulphate, polyethylene glycol or polyethyleneimine concentrations^11^, based on their pI, or in combination with affinity precipitation. Phase partitioning is based on the solubility of the proteins in regards to different liquid phases (e.g. aqueous two- or three-phase partition), but the resolution is still low to medium. These processes are very useful as the first steps of a protein purification method evaluation or if the final product is of low monetary value since high resolution methods are relatively more expensive. However, usually pharmaceutical proteins are purified utilizing high resolution methods based on chromatographic separations. Proteins are either separated based on charge (ion exchange), hydrophobicity (hydrophobic interaction chromatography (HIC)^12^ or reverse-phase high performance liquid chromatography (RP-HPLC), size exclusion chromatography (also called gel filtration), and binding (purification with a protein-tag or utilizing a binding region of the native protein)^13,14^. The current trend in protein production is to increase protein yields at every stage and to reduce the number of separation steps^10^.

Cell free protein synthesis (CFPS) has shown its importance for pharmaceutical protein production, including the industrial production of a commercial protein drug (rhGM-CSF)^15^. However, this product was still purified using traditional methods, such as weak anion exchange chromatography, followed by filtration and several size exclusion chromatography steps, with all their problems as stated before. Furthermore, rapid methods are needed for pharmaceutical protein synthesis and screening^16^, but so far simple and integrated workflows for protein synthesis, purification, bioconjugation, and formulation do not exist. Since each protein is unique, such a workflow should focus on the intended use of the protein^5^.

**Figure 1:**
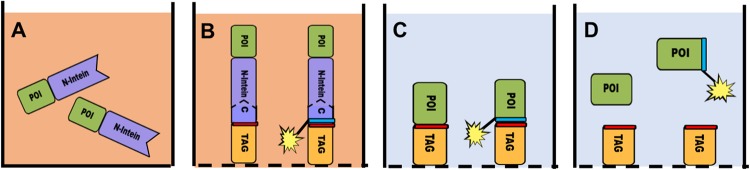
Schematic representation of the workflow. (**A**) Cell free protein synthesis (CFPS) of the protein of interest (POI) fused with an C-terminal split intein, typically 4–6 hours. The CFPS matrix is depicted in beige; (**B**) Split intein mediated capture, for 3 hours. The capture peptide is either in solution or immobilized to the surface (the dashed line depicts both scenarios). In orange the ‘Tag’ can be any affinity tag. The red bar is a photo-cleavable amino acid, while the blue bar is an unnatural amino acid linked to a reactive moiety. At this stage the intein will splice itself out spontaneously, without co-factors^46,47^; (**C**) After the intein reaction the CFPS matrix and side-products are washed away in under half an hour, and the final buffer is added (light blue); (**D**) after light triggered release (3 hours) POI is released into its final formulation. Bioconjugation can be performed now, later (for example *in-vivo*) or also at the end of step C before light treatment.

In this contribution, we demonstrate a workflow for the synthesis, purification, bioconjugation, and formulation of a pharmaceutical protein: human ciliary neurotrophic factor (hCNTF). Our approach utilizes cell free protein synthesis, split-intein mediated capture, light triggered release, moiety transfer upon release to facilitate bioconjugation, and formulating protein in the correct buffer at the desired protein concentration (see Fig. 1). This workflow omits many time-consuming steps of downstream protein production (e.g. the use of living cells, chromatography, dilution and re-concentrating, buffer exchange, purification of the bioconjugates, and re-folding). In principle, the proposed fast workflow can be performed in one day, instead of weeks that are spent using traditional methods. The proof-of-principle for this method is demonstrated here using ciliary neurotrophic factor (CNTF) and green fluorescent protein (GFP).

## Results

The following results are presented according to the workflow depicted in Fig. 1. However, several of the steps were also evaluated in together, in an integrated way.

### Cell free protein expression

Several cell-free systems were evaluated: *E. coli* BL21(DE3) S12 and S30 lysates (Figs S1 and S2), tobacco plant BY-2 cell lysates (BYL)^17^, commercial kits based on *E. coli* (Promega, USA; Figs S1 and S2), wheat germ (Biotechrabbit, Germany; Fig. S3), and mammalian HeLa cells (ThermoFisher scientific, USA). BYL and HeLa systems (both batch processes) gave the highest yields within 6 hours and were used for further evaluation.

**Figure 2:**
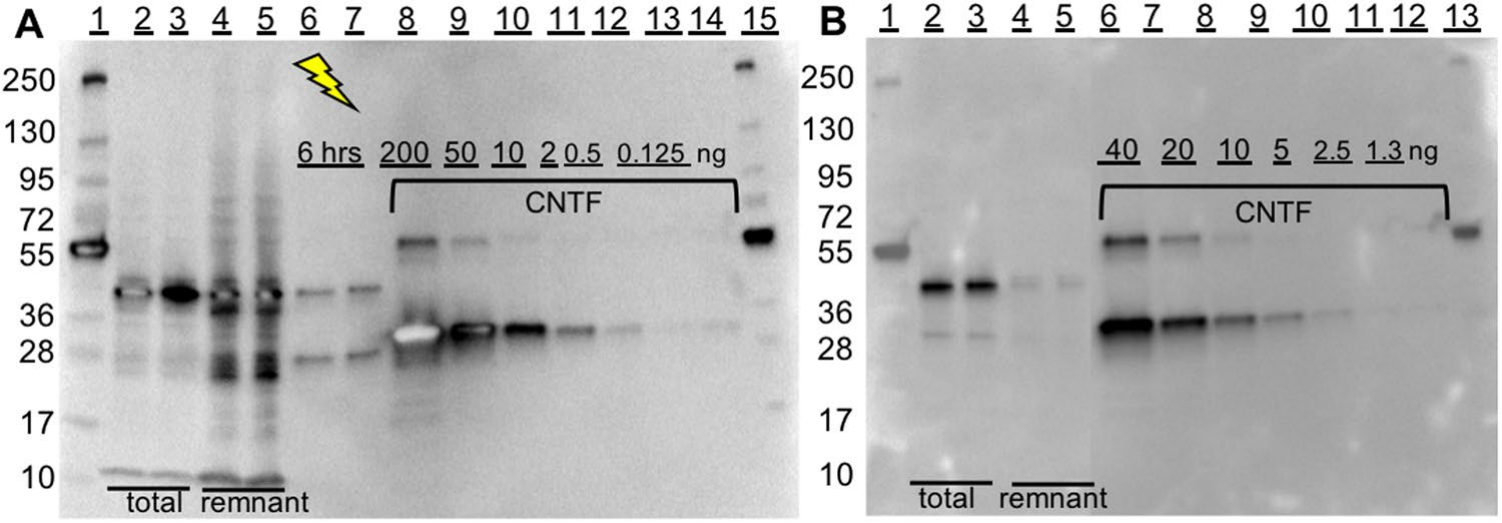
Quantitative analysis of the capture and release of hCNTF on the immobilized surface of magnetic beads. (**A**) WB of BYL based CFPS of hCNTF, lanes: (1&15): molecular weight marker; (2–3): total protein before split-intein mediated capture & intein reaction; (4–5): the remnant of the liquid fraction after immobilisation; (6–7): release of hCNTF after 6 hours of light triggered release (365 nm; 12.5 mW); (8–14): 200, 50, 10, 0.5, 0.125 ng of purified His_6_-hCNTF. (**B**) WB of HeLa cell lysate based CFPS of hCNTF, lanes: (1&13): molecular weight marker; (2–3): total protein before split-intein mediated capture & intein reaction; (4–5): the remnant of the liquid fraction after immobilisation; (6–12): 40, 20, 10, 2.5, and 1.3 ng of purified hCNTF. The uncropped images can be seen in Fig. S6.

Protein yields were determined from the western blots (WB; Fig. 2A,B) and are summarized in Table 1. Due to the limitations in the linear range of the WB-staining reagent the bands with highest concentrations were bleached out and therefore omitted from the quantitate analysis. The expression time in the BYL system was 16–20 hours at 25 °C, while hCNTF- *Npu*DnaE_ΔC16_ was expressed for 6 hours at 30 °C in the HeLa system. Overall, the amounts of hCNTF were 75% and 51% lower than the expected expression amounts of the positive controls of the BYL^17^ and the HeLa systems respectively (as given by ThermoFisher Scientific, USA). Please note that due to the absence of β-mercaptoethanol in the loading buffer for the SDS-PAGE, previously purified hCNTF appears as 2 bands on the WB. The major band is the monomeric and the minor band the dimeric form. In previous work Itkonen *et al*. in our laboratory have optimized the expression and purification of hCNTF^18^. Here we can observe one band, the monomeric form of purified hCNTF, in a SDS-PGE gel with β-mercaptoethanol in the Laemmli buffer, stained with Coomassie brilliant blue.

**Table 1:** Expression, capture, and light release efficiency of hCNTF with immobilization on the surface of magnetic beads.

CFPS system	Total amount hCNTF [mg/L]^a^	Amount hCNTF Captured beads [%]	Amount hCNTF Released^b^ [%]
BYL^c^	20.0	84	>6.3^e^
HeLa^c^	49.2	93	ND
HeLa^d^	ND	ND	>19^e^

^a^Milligram hCNTF per liter of CFPS matrix determined with hCNTF standard in the WBs; ^b^The percentages are based on the relative volumes of the bands of hCNTF released from the magnetic beads compared to the total synthesized hCNTF in the same WB gels; ^c^Immobilized peptide followed by split-intein mediated capture, washing, and light triggered release; ^d^split-intein mediated capture and protein *trans* splicing in solution followed by immobilization, washing, and light triggered release; ^e^corrected values are based on negative controls (Fig. S4). ND = Not Determined.

### Capture

The capture efficiency onto the beads in the CFPS matrixes was evaluated from the WBs before and after capture with peptides immobilized to magnetic beads from the relative amounts of hCNTF synthesized and remnant in the CFPS matrix (Table 1). The amounts of hCNTF bound to the beads were 0.42 μg/mg beads and 1.14 μg/mg beads for the BYL and the HeLa matrixes respectively. These values are an order of magnitude below the His_6_-POI binding capacity of the beads of >40 μg GFP/mg beads, indicated by the manufacturer.

### Purification

**Figure 3:**
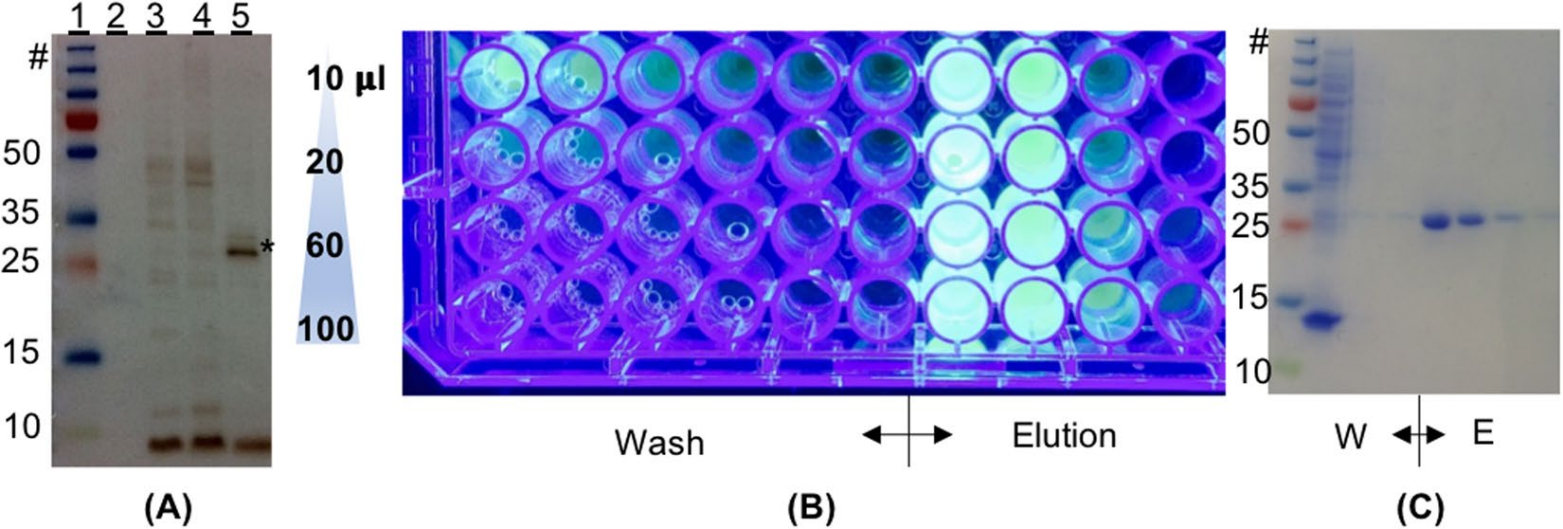
Expression, protein *trans* splicing, and purification of eGFP- *Npu*DnaE_ΔC16_ in *E. coli* prior to light triggered release. (**A**) WB using anti-polyhistidine antibody for the detection in the soluble fraction of *E. coli* lysate. Lanes: (1) molecular weight markers; (2) eGFP- *Npu*DnaE_ΔC16_ (not induced: negative control); (3) eGFP- *Npu*DnaE_ΔC16_ + peptide 1; (4) eGFP- *Npu*DnaE_ΔC16_ + peptide 3 + 1 mM ZnCl_2_; (5) eGFP- *Npu*DnaE_ΔC16_ + peptide 3 + 1 mM TCEP. (*) indicate the mass of eGFP-His6. (**B**) Elution of the soluble protein fractions after Ni-NTA affinity chromatography, using UV-light for visualization, of the products of the protein *trans* splicing of eGFP- *Npu*DnaE_ΔC16_ peptide 1. (**C**) SDS-PAGE stained with CBB of the first four washing steps and the elution steps. The uncropped images can be seen in Fig. S7.

In order to validate split-intein mediated capture and protein *trans* splicing in solution, eGFP- *Npu*DnaE_ΔC16_ (enhanced green florescent protein-*Npu*DnaE_ΔC16_) was expressed in *E. coli* cells as a recombinant fusion protein. The crude, soluble fraction of the cell lysate was split and either incubated with peptide 1 or peptide 3 to test the feasibility of capture and release in a complex matrix with high protein background, in this case cell lysate. Western blotting was utilized to visualize the target protein with anti-polyhistidine antibody (Fig. 3A). The products eGFP-His_6_ (hCNTF-(histidine)_6_) and eGFP-λ-His_6_ (hCNTF- photocleavable linker-(histidine)_6_) were purified using affinity chromatography (Fig. 3B,C). The final product eGFP, prepared without a His_6_ fusion tag, was clearly purified using affinity chromatography, demonstrating the feasibility of split-intein mediated capture and protein *trans* splicing in a complex matrix. The whole procedure, excluding the cloning experiments, was performed in 6 days.

**Figure 4:**
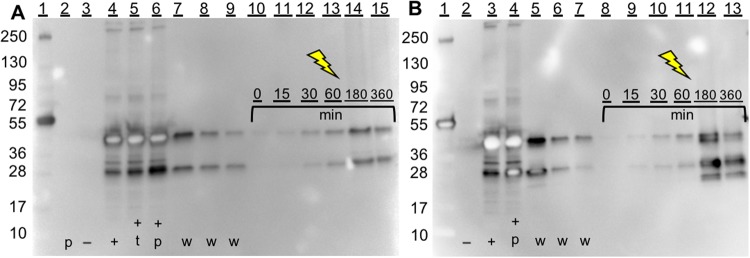
Removal of HeLa cell lysate based CFPS media from the solid support after expression and capture. (**A**) Split-intein mediated capture (split-intein mediated capture) after CFPS in reaction matrix before immobilization, followed by immobilization, washing, and light triggered release (light triggered release). Lanes: (1) molecular weight marker; (2) Peptide 3; (3) HeLa lysate without DNA (negative control); (4) hCNTF-*Npu*DnaE_ΔC16_ CFPS expression without TCEP; (5) hCNTF-*Npu*DnaE_ΔC16_ CFPS expression with 1 mM TCEP; (6) hCNTF- *Npu*DnaE_ΔC16_ after protein *trans* splicing with peptide 3; (7–9) wash steps 1, 2 and 3; (10–15) 0, 15, 30, 60, 180, and 360 minutes of light triggered release (365 nm, 12.5 mW). (**B**) Peptide immobilization followed by split-intein mediated capture, washing, and light triggered release. Lanes: (1) molecular weight marker; (2) HeLa lysate without DNA (negative control); (3) hCNTF-*Npu*DnaE_ΔC16_ CFPS expression without TCEP; (4) hCNTF- *Npu*DnaE_ΔC16_ CFPS expression after protein *trans* splicing with peptide 3; (5–7) wash steps 1, 2 and 3; (8–13) 0, 15, 30, 60, 180, and 360 minutes of light triggered release (365 nm, 12.5 mW). Uncropped images can be seen in Fig. S8.

The washing efficiency of captured and immobilized hCNTF from the Ni-NTA magnetic beads was determined in two scenarios: (a) split-intein mediated capture was performed in the BYL matrix follow by immobilization to Ni-NTA coated magnetic beads, and (b) split-intein mediated capture and protein *trans* splicing in the HeLa lysate matrix with prior immobilized peptides. Vigorous washing of the beads as instructed by the manufacture using the KingFisher (ThermoFisher Scientific) was equally effective for both scenarios and removed (50 ± 18) % of hCNTF from each previous washing step. Thus, before light triggered release, less than 1.5% of the remnant hCNTF was present. Vigorous washing with equal volumes are visualized in Fig. S4A (split-intein mediated capture and protein *trans* splicing with immobilized peptide in HeLa) and S4B (mock-split-intein mediated capture and mock-protein *trans* splicing in BYL). The washing steps depicted in Fig. 4 were less vigorous and concentrated before application to the SDS-PAGE gels in order to visualize if hCNTF was present in the washing step. After washing, hCNTF was cleaved off the beads via light triggered release (Fig. 4A,B) into the final buffer.

### Bioconjugation

**Figure 5:**
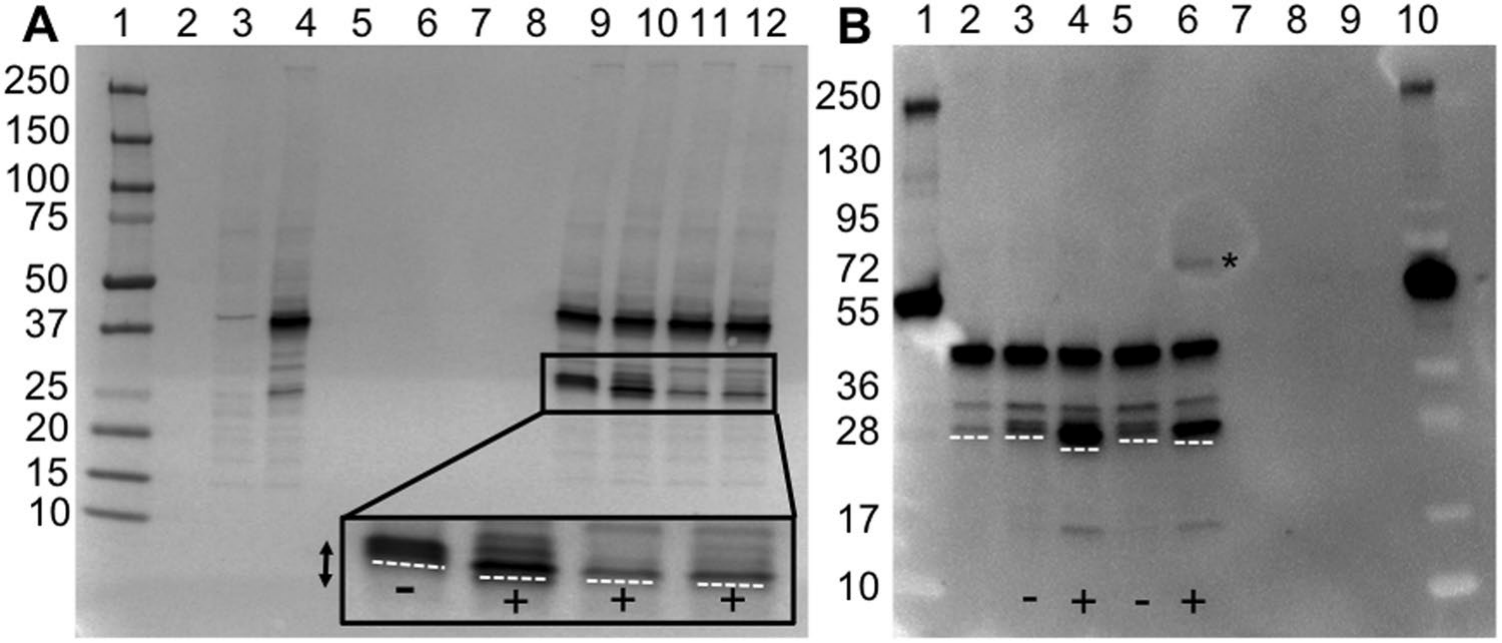
Transfer of biotin to hCNTF upon PTS. (**A**) Comparison of peptide 1 and peptides 5–7 (biotin-conjugated peptides). Lanes: (1) molecular weight marker; (2) Laemmli buffer; (3) HeLa lysate without DNA (negative control); (4) HeLa CFPS expressed hCNTF-*Npu*DnaE_ΔC16_; (5) Peptide 1; (6–8) Peptide 5–7; (9–12): hCNTF-*Npu*DnaE_ΔC16_ protein *trans* splicing with peptides 1, 5, 6, and 7 respectively. *Inset*: magnification of the hCNTF (−) and hCNTF-biotin bands (+). (**B**) Lanes: (1&10) molecular weight marker; (2) HeLa CFPS expressed hCNTF-*Npu*DnaE_ΔC16_ (total protein); (3) HeLa CFPS expressed hCNTF-*Npu*DnaE_ΔC16_ with peptide 1 (protein *trans* splicing reaction); (4) HeLa CFPS expressed hCNTF-*Npu*DnaE_ΔC16_ with peptide 5 (protein *trans* splicing reaction); (5) HeLa CFPS expressed hCNTF-*Npu*DnaE_ΔC16_ with peptide 1 (protein *trans* splicing reaction) and streptavidin; (6) HeLa CFPS expressed hCNTF-*Npu*DnaE_ΔC16_ with peptide 5 (protein *trans* splicing reaction) and streptavidin; (7–9) Laemmli buffer; (*) Streptavidin bound to biotinylated hCNTF. The white dashed lines indicate the front of the bands. Uncropped images can be seen in Fig. S9.

To demonstrate proof-of-principle of moiety transfer from the capture peptide to hCNTF, we utilized peptides 5–7 where biotin was conjugated to the peptides prior to split-intein mediated capture and protein *trans* splicing. Firstly, we transferred biotin to hCNTF synthesized in cell free matrix with peptides 5–7 within the same matrix and observed a migration-shift in the SDS-PAGE gels (Fig. 5A) where the hCNTF-biotin appears to be smaller, however, it migrates faster due its increased charge. This effect has been described earlier by Sano and Cantor (1990)^19^, where titration of streptavidin with biotin marked an increase in mobility on polyacrylamide gels.

In the streptavidin-shift assay, hCNTF-biotin was detected, though the relative amount appears to be much lower than the difference of hCNTF before and after incubation with streptavidin (Fig. 5B). In Fig. 5B we observe once again the higher mobility of hCNTF-biotin in lane 4, and possibly in lane 6.

After background correction, 18% of hCNTF is liberated after the split-intein mediated capture and protein *trans* splicing, relative to the amount of hCNTF- *Npu*DnaE_ΔC16_, with peptide 1 (Fig. 5B; lane 3), while peptide 5 is more efficient with 76% liberation of hCNTF. After incubation with streptavidin the relative amount of hCNTF in lane 5 did not change significantly (16%), while the amount of hCNTF-biotin in lane 6 compared to lane 4 is reduced by 56%. In comparison the amount of hCNTF-biotin-streptavidin, marked with an asterisk in lane 6, compared to the amount of hCNTF in lane 4 is 15%.

### Light triggered release

In order to evaluate the cleavage efficiency of the capture peptide’s photocleavable-linkers, the reduced peptides were incubated up to 30 minutes under UV light (365 nm; 12. mW) and analyzed with HPLC (Table 2; Fig. S5). The main peak of the chromatogram after 0 min of UV-light exposure represents 100% of non-cleaved peptide and its integrated peak area was compared to peaks at the same retention time of the same peptide. Peptide 1 and 5 do not contain a photocleavable moiety within the peptide structure, thus any observable decrease in the main peptide peak, and appearance of reaction product’s peaks in the chromatogram are solely due to the photocleavable moieties. The most efficient photocleavable group evaluated in this study is 3-(amino)-3-(2-nitrophenyl)propanoic acid (ANP)^20^, while 4-|4-[1-(9-fluorenylmethyloxycarbonylamino)ethyl]-2-methoxy-5-nitrophenoxy]butanoic acid (CAS 162827-98-7)^21^ is slightly more efficient than 2-nitrophenylalanine (F(2-NO_2_))^22^.

**Table 2:** Efficiency of light cleavage of peptides 1–3 and 5–7 determined with HPLC.

Peptide number	t_r_ of main peptide peak (min)	Cleavage efficiency (% of main peptide peak AUC left^a^) after UV-light exposure (365 nm; 12.5 mW) for:			
		0 min	5 min	15 min	30 min
1	9.975	100	~100	~100	~100
2	10.75	100	72	58	39
3	10.61	100	26	16	8
4	11.84	100	22	11	9
5	10.25	100	~100	~100	~100
6	11.10	100	41	38	30
7	11.80	100	43	39	25

^a^See also Fig. S5 for peptides 1–3.

We evaluated the photocleavage after (i) CFPS, (ii) split-intein mediated capture, and (iii) protein *trans* splicing in CFPS matrix followed by washing. Immobilization of hCNTF-λ-His_6_ on magnetic beads was also evaluated. hCNTF- *Npu*DnaE_ΔC16_ alone was not affected by light treatment (Fig. S4A), while light treatment of the hCNTF-λ-His_6_ liberated hCNTF (Figs 2A and 4). The release efficiency presented in Table 1 are conservative percentages. Due to technical limitations, the evaporation of the liquid from the mock-light triggered release samples (Fig. S4A), could not be compensated for, thus we overestimated the amount of hCNTF in the last lane of Fig. S4A of the negative control, resulting in a lower estimate of release of hCNTF in Figs 2A and 4. Hence, we can conclude that the release rates of hCNTF from the magnetic beads (5 mg beads/ml) is at least 6.3% if the peptide 3 is immobilized before split-intein mediated capture and protein *trans* splicing, while the release rate is at least 19% if immobilization occurs after split-intein mediated capture and protein *trans* splicing.

## Discussion

In summary, we demonstrated proof-of-principle of a fast work-flow to prepare, purify and bioconjugate a pharmaceutical relevant hCNTF protein. Cell free expression of proteins is rapid with yields high enough if followed by sensitive screening methods. Here we evaluated a plant-based system tobacco plant BYL and a mammalian-based system based on HeLa cells. The higher yield in the HeLa CFPS system (49 mg/L of hCNTF), compared to BYL was unexpected, but this may be due to the stability of hCNTF. The expression in the HeLa system was done in 6 hours, while the recommended time of 16–20 hours was applied for the BYL system^23^. Initially storage in a 100 mM NaH_2_PO_4_, 50 mM NaCl, 2 mM DTT buffer (pH 8.0) following expression of hCNTF in *E. coli*^18^ was not optimal, therefore a ThermoFluor assay^24^ was performed. From the thermo-ramping profiles, it was evident that hCNTF unfolds at 42–48 °C (data not shown). For comparison, hCNTF expressed in *E. coli* at a level of >112 mg/L of total protein using a codon optimized gene^18^. The *E. coli* expression and purification was done in 5–6 days.

The capture efficiency of hCNTF- *Npu*DnaEN_Δ16_ by the capture peptides within the CFPS matrix differed for both systems, mainly due to non-specific binding of proteins already present. This is supported by the negative control (Fig. S4B) where we observe native protein binding and elution to peptide coated Ni-NTA, a known issue when using Ni-NTA surfaces^25^. The capture efficiencies were determined by comparing the relative amounts of hCNTF before and after capture. Split-intein mediated capture onto magnetic beads in CFPS matrix was highly effective up to 93%. The presence of other proteins, therefore, does not seem to limit the capture efficiency. The capture efficiency of the split intein used, in buffer, extrapolated from Fig. S15 from Shah *et al*.^26^ appears to be 92–95%. In the same paper, the authors reported that the rate of the intein capture is lower at high salt concentrations. When compared to the specific binding capacities of His_6_-binding resins on magnetic beads, hCNTF is captured with both CFPS systems at a high to very high efficiency at similar bead concentrations^27^. However, the protein amounts bound via split-intein mediated capture followed by protein *trans* splicing are much lower than the capacity of the beads. In the BYL system we immobilized 0.42 μg hCNTF per mg of beads and for the HeLa system this was 1.14 μg hCNTF per mg of beads, while the binding capcity of the beads, according to the manufacturer is over 40 μg GFP per mg of beads. Since our relative binding efficiency is high, but the total amount of hCNTF is relatively low, it seems the maximal capacity of the magnetic beads functionalized with the capture- peptides was not reached.

In the EnBase^TM^ cultivation *E. coli* follows a linear growth curve^28^, the total protein background in the *E. coli* lysate was in a similar range of 75–100 fg protein per cell previously reported for slow dividing cells^29^. As shown in Fig. 3, eGFP-*Npu*DnaE_ΔC16_ treated with peptide 1 and 3, resulting in eGFP-His_6_ and eGFP-λ-His_6_, could be purified by use of Ni-NTA affinity chromatography. Therefore, we demonstrated that split-intein mediated capture and protein *trans* splicing in a complex and high protein background is possible in the crude, soluble fraction of *E. coli* lysate.

Following the capture and binding of hCNTF to Ni-NTA magnetic beads, 5 simple washing steps removed 98.5% of the remaining 7% of hCNTF. After demonstrating the photocleavability of the capture peptides (Table 2) with comparable values reported earlier^20–22^, we demonstrated their functionality in CFPS matrixes. Light triggered release was most effective when protein *trans* splicing was allowed in the complex CFPS matrix before immobilizing to magnetic beads where 19% of immobilized hCNTF was released from the beads due to light treatment. Compared to His_6_ fusion tags, coupled with Ni-NTA resin on magnetic beads, their elution (or release) efficiency is between 5–94% for 4 mg beads/ml^27^. Most likely an overcrowded peptide surface is less optimal for a functionally folded intein to form after split-intein mediated capture. During validation experiments of an earlier study (data not show) we found that for flat chitin-SPR sensors approximately 20% of chitin binding domain fused to *Npu*DnaE_ΔC16_ was an optimal distribution of the capture peptide^30^. One other limitation of the use of magnetic beads for light triggered release, is the difficulty to penetrate the magnetic bead slurry in the plastic wells of the KingFisher strips (ThermoFisher Scientific, USA) from above with UV-light. Furthermore, though the beads were mixed in a microtiter plate shaker throughout the UV-light exposure in a closed-box, the beads did sediment.

In addition to split-intein mediated capture, protein *trans* splicing, and light triggered release as an alternative protein purification protocol, we also wanted to demonstrate an alternative post-translational modification via intein mediated moiety transfer as an integrated part of the workflow, because bioconjugation is an important aspect of (biological) drug development^31–33^. As shown in the cartoon in Fig. 1, after protein *trans* splicing the POI is covalently linked to the capture peptide. We demonstrated that biotin was transferred from the capture peptide onto hCNTF in one step. In this reaction, no byproducts of the conjugation reaction needed to be removed prior to bioconjugation to the protein^34^. Furthermore, it appears that most of the hCNTF has been bioconjugated (Fig. 5A). Peptide 5 was utilized in a traditional streptavidin-shift assay^35^, since its split-intein mediated capture and protein *trans* splicing was more effective than with peptides 6 and 7 (Fig. 5B). In this reaction approximately 56% of hCNTF was biotinylated, however only 15% of that amount bound to streptavidin. The main reason for this low percentage is the over 100-fold excess of peptide 5 in the reaction mixture. This ‘captures’ a significant amount of the streptavidin away, which in turn cannot bind to the biotinylated hCNTF. In addition, the human anti-hCNTF antibody used for visualization might be less specific for the hCNTF-biotin-streptavidin complex than for hCNTF and hCNTF-biotin respectively. However, even though optimization is needed, moiety transfer after split-intein mediated capture and protein *trans* splicing is clearly demonstrated with cell-free protein synthesized hCNTF and in CFPS matrix.

The current limitations of the workflow depend on the ability to express the POI-intein fusion as a soluble protein and the efficiency of light cleavage while the captured protein resides on magnetic beads. Another current limitation is the yield in absolute amounts. CFPS is scalable^16^ and the amount captured is related to the total surface areas covered with the capture peptides. Even if protein production within a CFPS matrix is slower than expected, the CFPS reaction can be prolonged to capture the same amount over a longer period of time.

Currently we are evaluating this new work-flow on flat surfaces to optimize the light triggered release efficiency. We will couple this work-flow with process analytical techniques to evaluate the amount and quality of the pharmaceutical protein produced as well as in-line assays relevant to its function. Finally, we will indent to scale up the capture surface to increase the yields.

## Methods

### Materials

Unless specifically mentioned all commercial chemicals tris(2-carboxyethyl)phosphine (TCEP), ethylenediaminetetraacetic acid (EDTA), dithiothreitol (DTT), sodium azide (NaN_3_), triethanolamine hydrochloride (TEA), tris, sodium chloride, ampicillin, isopropyl β-D-1-thiogalactopyranoside (IPTG), magnesium chloride, imidazole, 2-(N-morpholino)ethanesulfonic acid (MES), and lysozyme were obtained from Merck Sigma-Aldrich (Darmstadt, Germany), Potassium chloride was obtained from Honeywell Riedel de Haën (Seelz, Germany), disodium hydrogen phosphate was obtained from Fisher Scientific (Hampton, USA). DNAse and RNAse were obtained from Epicentre (Madison, WI, USA). Phenylmethylsulfonyl fluoride (PMSF) was obtained from ThermoFisher Scientific, USA. Peptides were synthesized by Peptide Protein Research Ltd, Hampshire, U.K. Following reaction buffers and media were used: Buffer A: 100 mM MES, 500 mM NaCl, pH 7.0 (at 25 °C); Buffer B: 50 mM Tris/HCl, 50 mM NaCl, pH 8.0 (at 25 °C); Buffer C: 10 mM Tris/HCl, 150 mM NaCl, 1 mM TCEP, pH 7.4 (at 25 °C). EnPressoB medium was obtained from BioSilta Ltd. (U.K); currently available via EnPresso GmbL (Berlin, DE). Ultra-pure water, having a resistivity of 18 MΩ∙cm and a total organic carbon level of <5 ppm, prepared with a Milli-Q purification system was used for all measurements and for preparation of the buffers, the peptide solutions, and EnPressoB media.

### Construction of expression vectors

Yeast codon optimized human heat shock protein A1 (HspA1) was purchased from ATUM^SM^ (then DNA2.0, USA) and the HspA1 gene was cloned into the pIVEX1.4 Strep tag II – Tobacco Etch virus protease (TEV) plasmid by Biotechrabbit GmbH (DE). The pIVEX_GFP plasmid was purchased from Biotechnrabbit GmbH (DE) as component of an *E. coli* CFPS expression kit (order number BR1400101).

The codon optimized hCNTF gene was isolated from pOPINF-CNTF plasmid (Itkonen *et al*. 2014) in a gradient-PCR (57°–62 °C) with 5′-tttaagaaggagatatacatATGGCGTTTACCGAACATTC-3′ and 5′-gtttcatagctgaggcaCATCTTCTTGTTGTTTGCG-3′ as forward and reverse primers, while the eGFP gene was isolated from pEGFP-C1 plasmid (Clontech, USA) in a PCR with 5′- tttaagaaggagatatacatATGGTGAGCAAGGGCGAG-3′ and 5′-gtttcatagctgaggcaCTTGTACAGCTCGTCCATGC-3′ as forward and reverse primers. The NpuDnaE_N∆16_ harbouring pET21a-CBD-NpuDnaE_N∆16_ plasmid^30^ was linearized using 5′-TGCCTCAGCTATGAAACG-3′ and 5′-ATGTATATCTCCTTCTTAAAGTTAAAC-3′ as forward and reverse primers. The PCR products were assembled into plasmids pET21a-CNTF-NpuDnaE_N∆16_ and pET21a-eGFP-NpuDnaE_N∆16_ with NEBuilder®.

Plasmids pT7CFE1-CHis (Thermo Scientific, Rockford, IL, USA) and pIVEX-GAA_Omega_eYFP_His^17,23^ were used as vector backbones in constructing the expression plasmids for the CFPS of hCNTF-*Npu*DnaE_C∆16_ in the HeLa and Tobacco BYL CFPS systems, respectively. DNA fragments for the CFPS expression plasmids were generated as follows. The hCNTF- *Npu*DnaE_ΔC16_ sequence from the pET21-hCNTF-*Npu*DnaE_C∆16_ plasmid was amplified using gradient-PCR (57–62 °C) with 5′-gatgataatatggccaccacccatATGGCGTTTACCGAAC-3′ and 5′-tttttttt-ttttttttttttttCTAGCGCTCAACTCCAATGTC-3′ (Fragment A), and 5′-acattttacattctacaactacc-ATGGCGTTTACCGAACATTC-3′ and 5′-cagaagagctgggtaccttaTTAGCGCTCAACTCCAATG-3′ (Fragment B) as forward and reverse primers as for the HeLa and BYL constructs, respectively. The sequence harboring the C-terminal His_6_-tag was cloned out and the pT7CFE1-CHis plasmid linearized by restriction enzyme digestion with MscI and BglII (both NEB, MA, USA) (Fragment C). The sequence for eYFP-His was removed and the linearized pIVEX_GAA_Omega_ plasmid backbone amplified in an inverse gradient-PCR (64–69 °C) with 5′-TAAGGTACCCAGCTCTTC-3′ and 5′-GGTAGTTGTAGAATGTAAAA-TGTAATG-3′ as forward and reverse primers, respectively (Fragment D). Fragments A + C and B + D were assembled into plasmids pT7CFE1-hCNTF-*Npu*DnaE_ΔC16_ and pIVEX_GAA_Omega_hCNTF-*Npu*DnaE_ΔC16_, respectively, using NEBuilder® (NEB, MA, USA).

All DNA fragments were separated on 1% agarose gels, and bands of the appropriate sizes excised and cleaned up with NucleoSpin® Gel and PCR Clean-up columns (Macherey-Nagel, DE). The purified DNA fragments were assembled into plasmids using NEBuilder® HiFi DNA Assembly Cloning Kit (NEB, MA, USA) and the yielded expression plasmids were transformed into NEB-5α (NEB, MA, USA) *E. coli* competent cells^36^. Multiple colonies were picked and screened for the insertion of the hCNTF-*Npu*DnaE_ΔC16_ sequence by colony PCR using 5′-TAATACGACTCACTATAGGG-3′ and 5′-ttttttttttttttttttttttCTAGCGCTCAACTCCAATGTC-3′, and 5′-ATGTAATACGACTCACTATAGAAA-3′ and 5′-AGGTCCAAACCAAACCA-3′ as forward and reverse primers for HeLa and BYL constructs, respectively. Positive clones were propagated and the sequences of the amplified plasmids verified by gene sequencing (GATC Biotech, DE). Verified plasmid constructs were further amplified and purified to high concentration and purity using NucleoBond® Xtra Midi columns (Macherey-Nagel, DE). Plasmids were then transformed separately to *E. coli* strain BL21(DE3) (Invitrogen, USA) for protein production and in parallel stored at −20 °C for CFPS.

### Cellular protein expression and purification

Expression and purification of His_6_-hCNTF used for the controls was performed as previously described^18^. Briefly summarized, His_6_-hCNTF was produced in Rosetta 2 (DE3) pLysS with the auto-inducing media TBONEX (450 ml in 2000 ml baffled flask; 25 °C at 270 rpm for 30 h). Frozen cell pellets were freeze thawed and resuspended (3x) in 50 mM NaH_2_PO_4_, 300 mM NaCl, 10 mM imidazole, 1 mM DTT, protease inhibitor cocktail, and lysonase (pH 8.0). His_6_-hCNTF present in the soluble cell lysate fraction was purified with a Ni-IDA resin and size exclusion chromatography. The protocol had one adaption: a ThermoFluor assay^24^ was performed to find a buffer (buffer A) which provided a better His_6_-hCNTF stability than the original storage buffer. Expression of eGFP-His6-*Npu*DnaE_ΔC16_ was carried out in BL21(DE3) *E. coli* cells. First the transformants were grown o/n in LB Medium with 100 μg/ml ampicillin and 1% (w/v) glucose at 30 °C from glycerol stocks; 2 ml o/n culture was used to inoculate 50 ml of EnPressoB medium^28^ with 100 µg/ml ampicillin at 30 °C; 225 rpm (1″ amplitude shaker) according to manufacturer’s instructions in high yield flasks^37^. Cells were induced with 0.4 mM IPTG after 24 h and grown for another 24 h under the same conditions. The cells were separated from the media with centrifugation (16 000 * g for 15 min) and then lysed by freezing o/n at −20 °C in the presence of DNAse (2.5 U/ml), RNAse (2.5 U/ml), lysozyme (15 μg/ml), 0.1 mM PMSF (ThermoFisher Scientific, USA), and MgCl_2_ in buffer A. The insoluble fraction was removed by centrifugation (16000 g for 45 min). The crude, soluble *E. coli* lysate was kept on ice until further use. After incubating the cell lysates with either peptide 1 or peptide 3 Table 3) for 3 hours, the products of protein *trans* splicing were purified with a Protino® 96 Ni-NTA well plate with using the vacuum manifold (Macherey-Nagel, Germany) according to the manufactures instructions using buffer A and recommended imidazole concentrations. Different wash volumes were evaluated and performed (N = 6), elution was as instructed and was performed four-fold. The resulting protein fractions of the wash and eluate steps were visualized with SDS-PAGE gels using coomassie brilliant blue R-250 (ThermoFisher Scientific, USA)

**Table 3:** Photocleavable peptides used in this study.

Peptide number	*Npu*DnaE_C16_ peptide sequence with non-canonical amino acid/ conjugation in bold	Non-canonical amino acid/conjugation	Peptide sequence introduced on C-terminus of POI via PTS
1	DGHNFALKNGFIASNCFGSKHHHHHH	N/A	CFGSKHHHHHH
2	DGHNFALKNGFIASNC**F(2-NO**_**2**_)GSKHHHHHH	F(2-NO_2_): 2-Nitrophenylalanine^22^	C**F(2-NO**_**2**_)GSKHHHHHH
3	DGHNFALKNGFIASNC-**ANP**-GSKHHHHHH	ANP: 3-(Amino)-3-(2-nitrophenyl)propionic acid^20^	C-**ANP**-GSKHHHHHH
4	DGHNFALKNGFIASNCF-**X**-GSKHHHHHH	X: Photolinker CAS 162827-98-7^21^	CF-**X**-GSKHHHHHH
5	DGHNFALKNGFIASNCGSG-**X**-GGHHHHHH	X: Biotin on side chain of Lysine	CGSG-**X**-GGHHHHHH
6	DGHNFALKNGFIASNCGSG-**XY**-GGHHHHHH	X: Biotin on side chain of Lysine Y: Photolinker CAS 162827-98-7^21^	CGSG-**XY**-GGHHHHHH
7	DGHNFALKNGFIASNCGSG-**XY**-GGEPEA	X: Biotin on side chain of Lysine Y: Photolinker CAS 162827-98-7	CGSG-**XY**-GGEPEA

### Cell free protein synthesis (CFPS)

*E. coli* BL21(DE3) S12 and S30 lysates^38–41^, tobacco plant or BYL^17^, *E. coli* (Promega, USA, order number L1110), wheat germ (Biotechrabbit, Germany, order number BR1401001), and mammalian HeLa cells (ThermoFisher Scientific, USA, order number 88881) were utilized for protein expression according to the published protocols or manufacturer’s instructions. The quality of each CFPS reaction was checked by use of a positive control protein (GFP) in parallel to the CFPS reactions of the samples. Expression levels were also compared to a negative control where no DNA was added to the reaction. Continuous-exchange cell-free expression (CECF) of Strep-Tag II _TEV- HspA1 was performed in wheat germ lysates. CFPS of GFP was performed in *E. coli* BL21(DE3) S12 and S30 lysates with the PremixPlus reaction mixture from the Promega *E. coli* kit and *E. coli* lysate (Promega, USA) with the PremixPlus, the ‘Swartz’ reaction mix^41^, and the ‘EMBL’ reaction mix^39^. CFPS of hCNTF-*Npu*DnaE_ΔC16_ was carried out in the BYL and HeLa systems. Briefly, required reaction components were mixed, plasmid DNA template was added and the reaction mixtures incubated accordingly; BYL CFPS reactions were incubated in an incubator-shaker (25 °C for 20 h) in 96-well plates while HeLa CFPS reactions took place at 30 °C during 6 h in 1.5 ml microcentrifuge tubes. The synthesized proteins in the CFPS matrix were used in subsequent experiments immediately.

### Capture and Release

The validity of the intein-mediated protein *trans* splicing of the artificially split *Npu*DnaE intein (*Npu*DnaE_ΔC15_/*Npu*DnaE_C15_^42,43^; see availability of data and materials) in the capture and release of expressed protein was studied. The expressed, captured and released hCNTF was studied with Western Blotting. To verify that protein *trans* splicing can indeed ligate a C-terminal peptide sequence with e.g. a photocleavable moiety and a His_6_-tag (for the capture and release) to the expressed POI, the following experiments were set up. PTS reactions between photocleavable peptide 2/peptide 3 (Table 3) and CFPS reactions containing expressed hCNTF-*Npu*DnaE_C∆16_ were carried out for 3 hours at room temperature. The resulting putative His_6_-tagged hCNTF was captured with a 1 mg of HisPur™ Ni-NTA Magnetic Beads, corresponding to a 5–10 mg/ml final concentrations of beads (ThermoFisher Scientific, USA). All handling of the magnetic beads was carried out with the KingFisher™ Purification System (ThermoFisher Scientific, USA). The unbound proteins and components of the CFPS reactions were subsequently removed from the beads by five washing-steps with 100 μl 0.05% Tween-20 in PBS (pH 8.0). Release of hCNTF was carried out by photocleavage with UV light (365 nm; 12.5 mW) for 0–360 min.

The feasibility of utilizing the intein ligation reaction itself in the capture and subsequent release of the expressed protein was also studied. In this setup, peptide 3 was first immobilized on the Ni-NTA beads. Intein ligation reaction between immobilized peptide 3 and CFPS expressed hCNTF- *Npu*DnaE_ΔC16_ was carried out overnight in room temperature, resulting in the ligation product, hCNTF-His_6_, that remains immobilized on the magnetic beads. After removal of unbound proteins and CFPS reaction components by wash steps, release of hCNTF was carried out by photocleavage with UV light (365 nm; 12 mW) for 0–360 min.

To verify that the capture and release were indeed achieved *via* the intein ligation reaction, control experiments were carried out. To rule out nonspecific binding to the Ni-NTA beads, a mock binding experiment was carried out using only the CFPS expressed hCNTF- *Npu*DnaE_ΔC16_ with the beads. After removal of unbound proteins and components of the CFPS reaction by wash steps, ‘release’ was carried out by photocleavage with UV light (365 nm; 12.5 mW) for 0–360 min. To verify the release having taken place *via* cleavage of the photolabile unnatural amino acids, the non-photocleavable peptide 1 was first immobilized on the Ni-NTA beads. Intein ligation reaction between immobilized peptide 1 and CFPS expressed hCNTF- *Npu*DnaE_ΔC16_ was carried out overnight in room temperature, resulting in the ligation product, hCNTF-His_6_, staying immobilized on the magnetic beads. After removal of unbound proteins and components of the CFPS reaction by wash steps, ‘release’ of hCNTF was carried out by photocleavage with UV light (365 nm; 12.5 mW) for 0–360 min.

### Bioconjugation

Protein *trans* splicing reactions were carried out between expressed hCNTF- *Npu*DnaE_ΔC16_ and *Npu*DnaE_C16_ peptide 1 or modified peptides 5, 6 or 7; the biotinylated peptide 5, peptide 6 and peptide 7 were used to assess if the respective modifications (Table 3) affect the Protein *trans* splicing e.g. through steric hindrance, while the non-conjugated peptide 1 was used as a positive control for the reaction. Briefly, hCNTF- *Npu*DnaE_ΔC16_ in HeLa CFPS reaction matrix was mixed with peptide 1, peptide 5, peptide 6 or peptide 7 (all 100 µM; reduced with 25 mM TCEP at 27 °C for 30 min) in buffer C and the PTS allowed to take place in room temperature for 2 h. Reactions were halted with the addition of 4X Laemmli sample buffer.

In a similar setup, peptide 5 was used to assess transferring biotin onto the protein of interest *via* PTS, while peptide 2 was used as a negative control. PTS reactions were set up as described earlier and carried out at room temperature overnight. Taking advantage of the well-documented strong and SDS-resistant binding between streptavidin and biotin, a streptavidin gel-shift assay (adapted from Fairhead and Howarth and Sorensen *et al*.^35,44^) was used to confirm transfer of biotin moiety to hCNTF; the described PTS reaction setups yielding the putative non-biotinylated and biotinylated hCNTF products were mixed with streptavidin in PBS and the streptavidin-biotin binding allowed 3 hours in room temperature. Detection of hCNTF biotinylation and subsequent streptavidin binding was carried out by Western Blotting.

### HPLC

To study the photocleavage efficiency and rate of synthesized *Npu*DnaE_C16_-peptides with varying linkers and conjugates, the peptide samples were dissolved in ultra-pure water and reduced with 25 mM TCEP at 27 °C for 30 min and then exposed to UV-light (365 nm; 12.5 mW) for 0–30 min. The generated peptide traces were separated with an Agilent 1100 HPLC system (Agilent Technologies, Palo Alto, CA) with UV detection at 216 nm. A Discovery® BIO Wide Pore C18 (150 × 4.6 mm, 5 µm; Sigma-Aldrich) column maintained at 25 °C and at a flow rate of 1.5 ml/min was used for all analyses. Samples (15 µl) were injected into the column using water (A) and ACN (B), both with 1% TFA, as eluents. The gradient program (0–14 min 1 → 40% B, 14–16 min 40 → 70% B, 16–16.1 min 70 → 1% B, followed by 14 min equilibrium at 1% B) was used to elute all peptide samples. Resulting chromatograms were examined and processed with ChemStation software A.08.03 (Agilent Technologies); decrease in the AUC of the respective main peptide peak was used as a surrogate to measure light triggered cleavage of the different peptides.

### Western blotting

Protein samples were resolved on precast 4–20% Mini-Protean® TGX Stain-Free SDS-PAGE gels (Bio-Rad) with PageRuler™ Prestained Protein Ladder (ThermoFisher Scientific, USA) or Precision Plus Protein™ WesternC™ Standard (Bio-Rad, USA) used as molecular weight markers. Separated proteins were transferred onto 0.2 µm nitrocellulose membranes with the Trans-Blot® Turbo™ Transfer System (Bio-Rad) according to manufacturer’s instructions and the membranes blocked in 2% bovine serum albumin (BSA) in Tris-buffered saline, 0.05% Tween 20 (TBS-T) for 2 h in room temperature or overnight in +4 °C. Proteins were detected in a sandwich reaction with 0.1–0.2 µg/ml rabbit anti-hCNTF polyclonal antibody (ThermoFisher Scientific, USA) and 0.1 µg/ml horseradish peroxidase-conjugated goat anti-rabbit IgG (Merck Sigma Aldrich, DE) as primary and secondary antibodies, respectively. Staining was carried out with Amersham™ ECL™ Prime Western Blotting Detection Reagent (GE Healthcare, USA) and the chemiluminescence detected with ChemiDoc™ XRS+ imaging system (Bio-Rad, USA). The band intensities were quantified using with the Image Lab™ software (Bio-Rad, USA)^45^. In brief, we image the WB with UV light and subtract the background after inspecting each lane. Known hCNTF quantities were used to calculate the amount of hCNTF on the sample gels. Overexposed band were excluded from the quantitative analysis. Membranes were rinsed with TBS-T between incubations.

### Availability of data and materials

The datasets supporting the conclusions of this article are included within the article and the supplementary data file.

The intein nomenclature is according to Aranko *et al*.^46^, however since our capture peptides are 16 amino acids long, we use *Npu*DnaE_ΔC16_ instead of *Npu*DnaE_ΔC15_, however the split-site is the same.

## Electronic supplementary material

Supplementary figures S1-S9
