# Supplementary material for: Accelerated pharmaceutical protein development with integrated cell free expression, purification, and bioconjugation
Source: Sci Rep. 2018 Aug 10;8:11967. doi: 10.1038/s41598-018-30435-4 (PMC6086869; doi:10.1038/s41598-018-30435-4)
Supplement: Supplementary file 1 — Supplementary figures S1-S9 [file 41598_2018_30435_MOESM1_ESM.docx]

**Accelerated pharmaceutical protein development with integrated cell free expression, purification, and bioconjugation**

Dominique Richardson^1§^, Jaakko Itkonen^1§^, Julia Nievas^1,2^, Arto Urtti^1,3,4^, Marco G. Casteleijn^#1^

**Supplementary data**

**Cell Free Protein Expression**

Cell free protein expression in *E. coli* lysates and wheat germ lysate and their relative yields are depicted in figures S1-S3.

Figure S2.

**Figure S1.** Expression levels of green florescent protein (GFP) in relative fluorescent units (RFU) followed over time with different final CFPS reaction mixtures. GFP was excited at 395 nm and emissions were recorded at 504 nm. Here, pIVEX_GFP refers to the genetic construct containing the GFP gene, expressed after induction under the T7 promoter [1], [S12 [2] + beads] and [S30 [3] + sonication] refer to the method of cell lysate preparation [4]. The Swartz CFPS mixture [5] and the EMBL mixture [6] refer to composition of the reaction-mixture in the final CFPS reaction. BL21(DE3) cells were purchased from Novagen (USA), now Merck-Millipore (DE).

**Figure S2.** Expression levels of green florescent protein (GFP) in relative fluorescent units (RFU) followed over time with different final CFPS reaction mixtures. GFP was excited at 395 nm and emissions were recorded at 504 nm. Here, pIVEX_GFP refers to the genetic construct containing the GFP gene, expressed after induction under the T7 promoter [1], [S12 [2] + beads] and [S30 [3] + sonication] refer to the method of cell lysate preparation [4]. The Swartz CFPS mixture [5] and the EMBL mixture [6] refer to composition of the reaction-mixture in the final CFPS reaction. BL21(DE3) cells were purchased from Novagen (USA), now Merck-Millipore (DE).


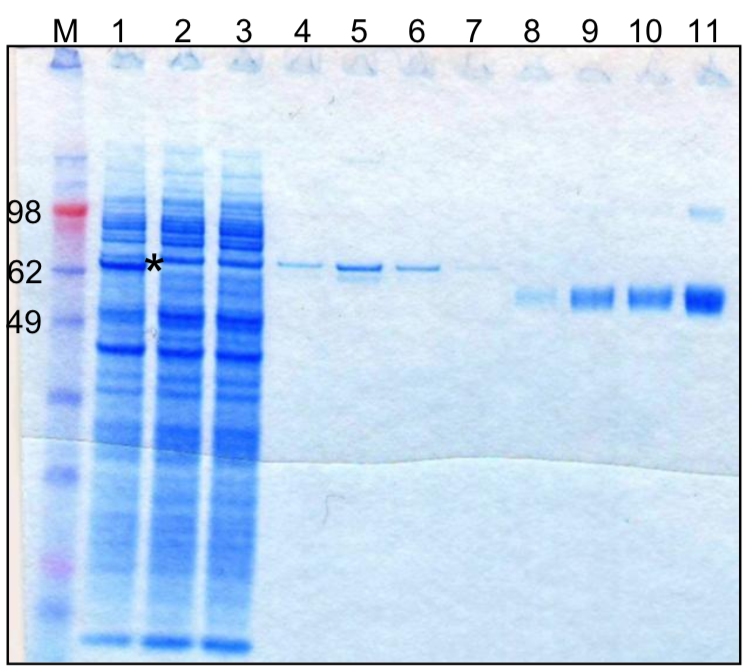


**Figure S3.** Protein synthesis with wheat germ lysate based continuous-exchange cell-free, or CECF [7], of StrepTag II – TEV - HspA1 (*). SDS-PAGE gel of the expression and purification, with BSA at concentrations of 0.2 / 0.5 / 1.0 / 1.5 μ (lanes 8 – 11) for comparison; M: molecular weight marker; 1: positive control Beta-glucuronidase (~ 68 kDa); 2: StrepTag II – TEV - HspA1, total protein (~72 kDa); 3: StrepTag II – Tev - HspA1, soluble protein (~72 kDa); 4 – 7: StrepTag II – TEV - HspA1 eluate fractions (5 μl).

**Negative controls**

Figure S4A indicates that some CNTF protein is released off the magnetic Ni-NTA beads despite the absence of a photo-cleavable linker, while figure S4B shows that CNTF-*Npu*DnaE_C∆16_ doesn't by itself bind to beads, nor is there release of native CNTF from the beads.

**Figure S4.** Negative controls of binding and LTR after immobilizing on magnetic Ni-NTA beads. (A) Peptide 1 (non-photo-cleavable peptide) immobilization followed by SIMC, washing, and “light triggered release”. Lanes: (1): molecular weight marker; (2) BY-2 CNTF-*Npu*DnaE_C∆16_ expression; (3-5): washes 1, 3 and 5 after peptide 1 immobilization; 6: SIMC with Ni-NTA immobilized peptide 1 remnant; 7-9: washes 1, 3 and 5; 10-15: “light triggered release” for 0, 15, 30, 60, 180 and 360 min (12.5 mW, 365 nm). (B) Lanes: (1): molecular weight marker; (2) BY-2 no DNA (negative controle); (3) CNTF-*Npu*DnaE_C∆16_ BY-2-CFPS expression; (4) BY-2 CFPS expressed CNTF-*Npu*DnaE_C∆16_  in absence of peptide (mock protein *trans* splicing); (5) Remnant of CNTF-*Npu*DnaE_C∆16_ capture on magnetic Ni-NTA beads; (6-8) wash steps 1, 3 and 5; (9-13): 0, 15, 30, 60, and 180 minutes of ‘LTR’ (365 nm, 12.5 mW). (C) uncropped full WB image of S4A. (D) uncropped full WB image of S4B. The exposure time of S4A/C is 2.04 sec, and for S4B/D 2.71 sec.

**HPLC chromatograms**


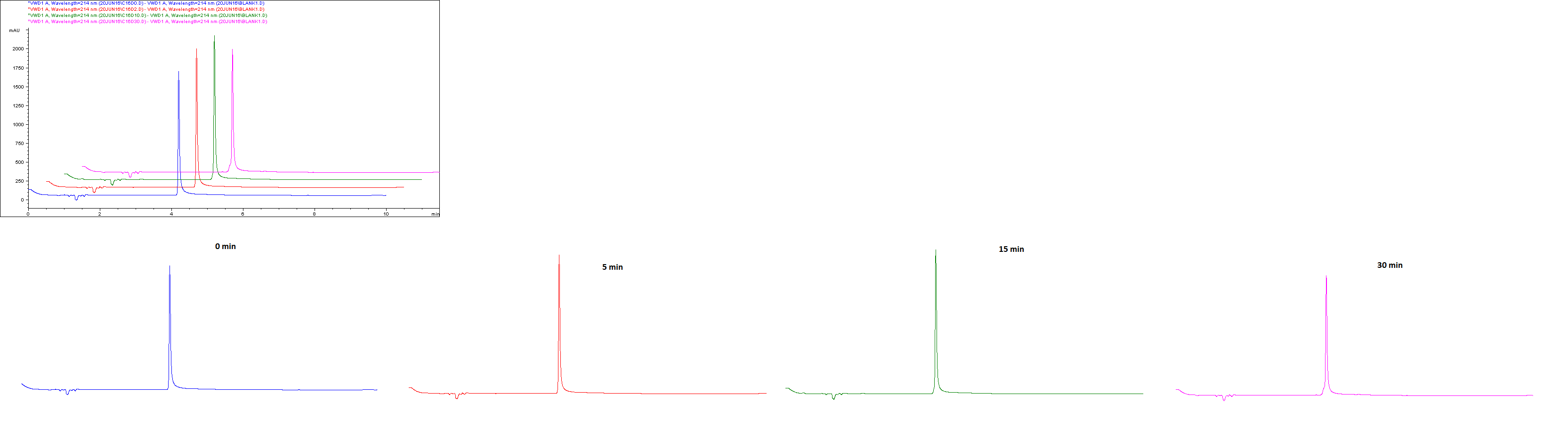


**A**


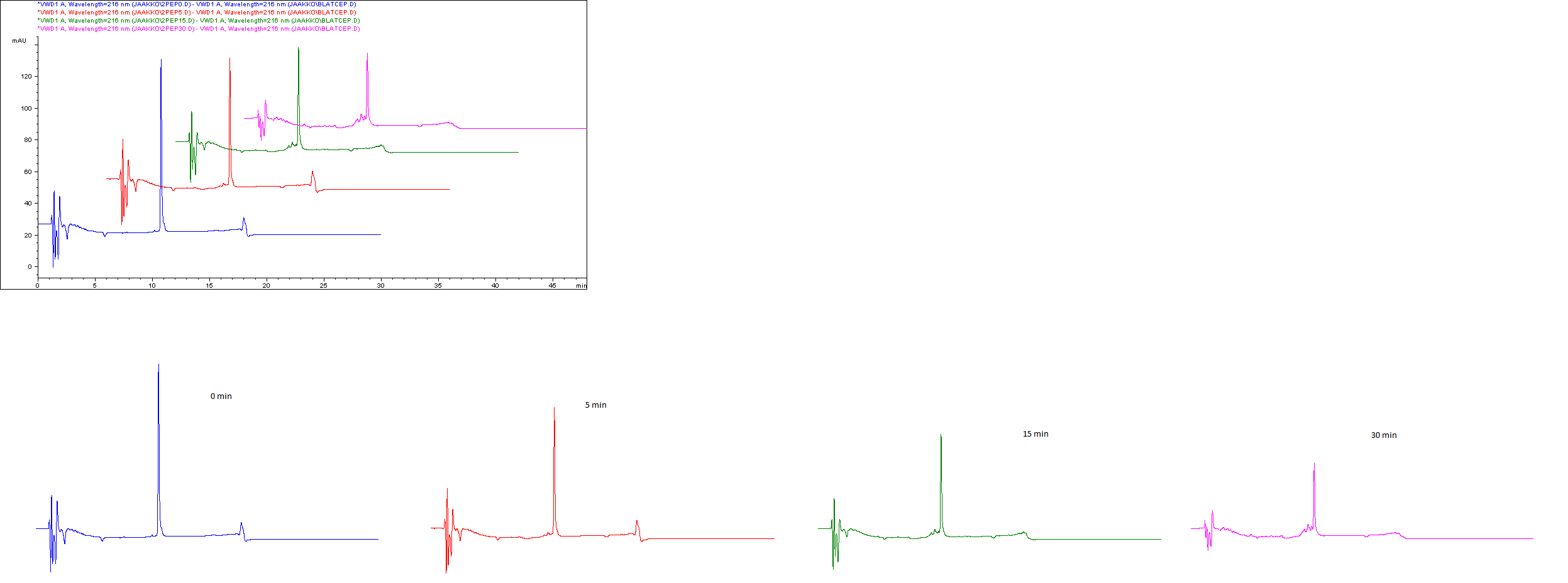


**B**


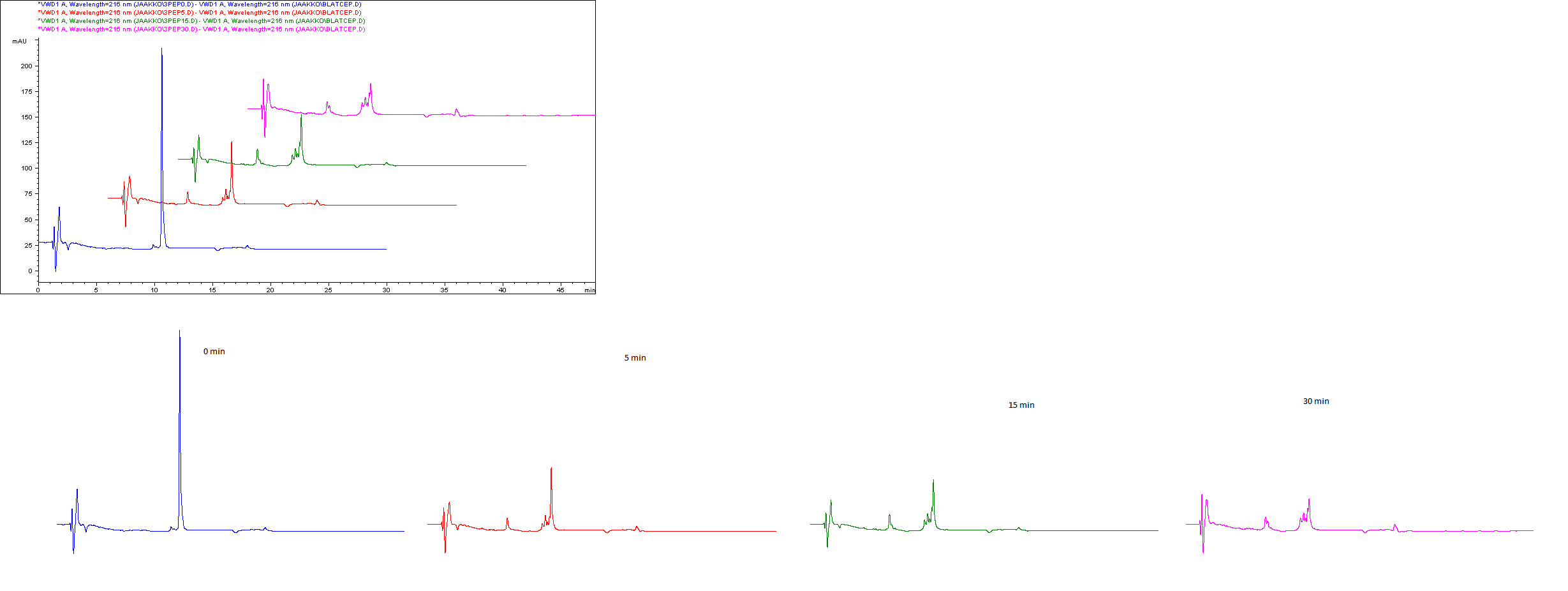


**C**

**Figure S5.** HPLC chromatograms of (A) peptide 1, (B) peptide 2, and (C) peptide 3 in aqueous solution after 0 (blue), 5 (red), 15 (green), and 30 (magenta) minutes of light cleavage at 12.5 mW and 365 nm.

**Uncropped images**

**Figure S6.** Uncropped WBs as used for figure 2 in the main text. (A) as in figure 2A; exposure time was 0.40 sec. (B) as in figure 2B, lanes 6 and 7 were empty and cropped out in figure 2B; exposure time was 2.00 sec.

**Figure S7.** Uncropped SDS-PAGE gels and 96 well plate as used for figure 3 in the main text. (A) as in figure 3A; lanes 6 – 9 were loaded with alternative variants of *Npu*Dna split inteins not used in this paper, lanes 10-15 were not loaded and cut before imaging. (B) as in figure 3B, top 4 rows now show a duplicate purification, while the two columns of the left were not used. (C) as in figure 3B; lanes 9-15 show a duplicate expression, wash and elution.

**Figure S8.** Uncropped WBs as used for figure 4 in the main text. (A) as in figure 4A; exposure time was 1.59 sec. (B) as in figure 4B, lanes 14 and 15 were cropped off: lane 14 was loaded with poorly resolved molecular weight marker; lane 15 was empty; exposure time was 1.74 sec.

**Figure S9.** Uncropped WBs as used for figure 5 in the main text. (A) as in figure 5A, with lanes 13-15 empty and cropped off in figure 5A; exposure time was 0.15 sec. (B) as in figure 5B, with lanes 11-15 empty and cropped off in figure 5B; expose time was 5.70 sec.

**References:**

1. Roge J, Betton JM: **Use of pIVEX plasmids for protein overproduction in Escherichia coli.** *Microb Cell Fact* 2005, **4:**18.

2. Kim TW, Keum JW, Oh IS, Choi CY, Park CG, Kim DM: **Simple procedures for the construction of a robust and cost-effective cell-free protein synthesis system.** *J Biotechnol* 2006, **126:**554-561.

3. Kigawa T, Yabuki T, Matsuda N, Matsuda T, Nakajima R, Tanaka A, Yokoyama S: **Preparation of Escherichia coli cell extract for highly productive cell-free protein expression.** *J Struct Funct Genomics* 2004, **5:**63-68.

4. Shrestha P, Holland TM, Bundy BC: **Streamlined extract preparation for Escherichia coli-based cell-free protein synthesis by sonication or bead vortex mixing.** *Biotechniques* 2012, **53:**163-174.

5. Jewett MC, Calhoun KA, Voloshin A, Wuu JJ, Swartz JR: **An integrated cell-free metabolic platform for protein production and synthetic biology.** *Mol Syst Biol* 2008, **4:**220.

6. **Protein Expression. E.COLI: HOW TO PREPARE YOUR OWN CELL-FREE SYSTEM** [<https://www.embl.de/pepcore/pepcore_services/protein_expression/ecoli/lysate/index.html#setting>]

7. Spirin AS, Baranov VI, Ryabova LA, Ovodov SY, Alakhov YB: **A continuous cell-free translation system capable of producing polypeptides in high yield.** *Science* 1988, **242:**1162-1164.
